# One-Stage Surgical Management of Adult Native Coarctation and Severe Aortic Stenosis: A Case Report

**DOI:** 10.3390/jcdd13050203

**Published:** 2026-05-09

**Authors:** Dejan M. Lazovic, Milica Karadzic Kocica, Stefan Juricic, Dragan Ivanisevic, Vojkan Aleksic, Mladen J. Kocica, Danko Grujic, Jovana Klac, Jovana M. Mihajlovic, Vladimir Jovicic, Dragan Cvetkovic

**Affiliations:** 1 Clinic for Cardiac Surgery, University Clinical Center of Serbia, 8th Kosta, Todorović St., 11000 Belgrade, Serbia; draganivanisevic0503@gmail.com (D.I.); valeksicc@gmail.com (V.A.); kocica@sbb.rs (M.J.K.); danko0702@gmail.com (D.G.); medi@eunet.rs (V.J.); dcgoje@gmail.com (D.C.); 2 Faculty of Medicine, University of Belgrade, 11000 Belgrade, Serbiajoantheoberste@gmail.com (J.M.M.); 3 Center for Anesthesiology, Reanimatology and Intensive Care Medicine, University Clinical Center of Serbia, 8th Kosta Todorović St., 11000 Belgrade, Serbia; 4 Clinic for Cardiology, University Clinical Center of Serbia, 8th Kosta Todorović St., 11000 Belgrade, Serbia; stefan.juricic@gmail.com (S.J.); jovanablagojevic718z@gmail.com (J.K.)

**Keywords:** coarctation, extra-anatomic, aortic valve replacement, aortic stenosis

## Abstract

The coarctation of the aorta is a congenital anomaly characterized by a local narrowing of the aortic lumen localized near the ductus arteriosus. Typically diagnosed in childhood, but it can remain until symptoms become evident. This aortic anomaly can also coexist with aortic valve stenosis. In our case report, we present a 46-year-old male with chest pain, dyspnea, and a significant blood pressure gradient between upper and lower extremities. Diagnostic examination included transthoracic echocardiography and computerized tomography. This diagnostic imaging showed narrowing of the aortic lumen with a residual lumen dimension of 3 mm and severe aortic stenosis. The patient underwent a complex surgical procedure, replacement of the aortic valve and reconstruction of the aorta. An extra-anatomic ascending-to-descending aortic bypass was constructed using a 20 mm Dacron graft, combined with mechanical aortic valve replacement. The operation was performed through median sternotomy with two arterial canula in the femoral artery and in the aorta, and one venous canula in the right atrium. Two canulae are placed for the safe performance of cardiopulmonary bypass. The patient was discharged at home without complication. This case highlights that a single surgical procedure may represent a definitive treatment of a complex problem with good short-term results.

## 1. Introduction

Coarctation of the aorta (CoA) is a congenital cardiovascular anomaly. It presents as a narrowing of the aortic lumen, most commonly caused by a constriction at the site of the ductus arteriosus or ligamentum arteriosum [1]. The histological composition of the ductus arteriosus—longitudinal smooth muscle fibers—contrasts with the circumferential elastic fibers of the aorta. This structural difference facilitates the normal postnatal closure of the ductus arteriosus but may also contribute to aortopathies later in life [2]. Survival into adulthood despite this pathology highlights its significant clinical consequences [3]. Comorbidities and underlying pathologies involving left ventricular dysfunction and hemodynamic consequences (hypertension, aneurysms, cerebrovascular events, etc.) are frequent [4].

Coarctation of the aorta accounts for approximately 4–7% of all congenital heart diseases and occurs more frequently in males than in females, with a male-to-female ratio of 2:1 [5]. About 10% of patients remain asymptomatic and are diagnosed after the age of 50 [6]. While this statistic holds academic value, it is important to consider that prevalence rates may vary based on lifestyle factors or healthcare accessibility, which only emphasizes the socioeconomic and technological disparities across regions. In this particular case, the narrowing of the aorta was accompanied by aortic valve stenosis, presenting a complex and clinically significant combination.

## 2. Case Presentation

**Figure 1:**
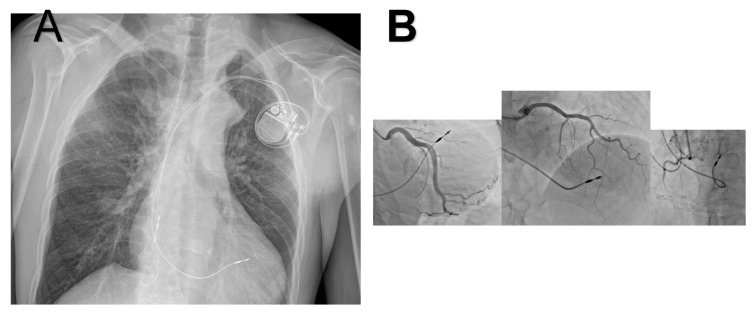
(**A**) Chest X-ray of thorax and (**B**) coronary angiography.

We present the case of a 46-year-old man who was admitted to our Cardiac Surgery Clinic due to intense fatigue, chest pain, difficulty breathing, as well as occasional pain in the lower extremities during minimal effort. The patient had a history of this intense symptoms for the past 9 months. The patient was fully clinically, laboratorially and radiologically examined preoperatively (Figure 1A).

During the physical examination, the pressure on the right radial artery was 170/100 mmHg, and on the right femoral artery was 100/45 mmHg. In laboratory analyses, prerenal kidney insufficiency presents with creatinine 160 mmol/L, urea 11.2 mmol/L, and glomerular filtration 50 mL/min/1.73 m^2^. Other laboratory parameters were normal. Additionally, the diagnostic tests showed the following: Electrocardiography marked left ventricular hypertrophy (pronounced R wave); and auscultation with a stethoscope showed a precordial systolic murmur. Posterior systolic murmur suggestive of collateral circulation was detected on physical examination. Transthoracic echocardiography confirmed the presence of significant aortic stenosis with a bicuspid aortic valve type I (a gradient of approximately 50 mmHg is registered over the described coarctation). Hemodynamically significant aortic stenosis was documented with a peak gradient (PG) 51 mmHg, mean gradient (MG) 39 mmHg, aortic valve area (AVA) 0.9 cm^2^, indexed aortic valve area (AVAi) of 0.6 cm^2^, stroke volume (SV) of 50 mL, and stroke volume index (SVI) 30 mL/m^2^/BSA, associated with trace aortic regurgitation. Selective coronary angiography was also performed, which showed that there are no hemodynamically significant changes in the coronary vessels of the heart (Figure 1B).

**Figure 2:**
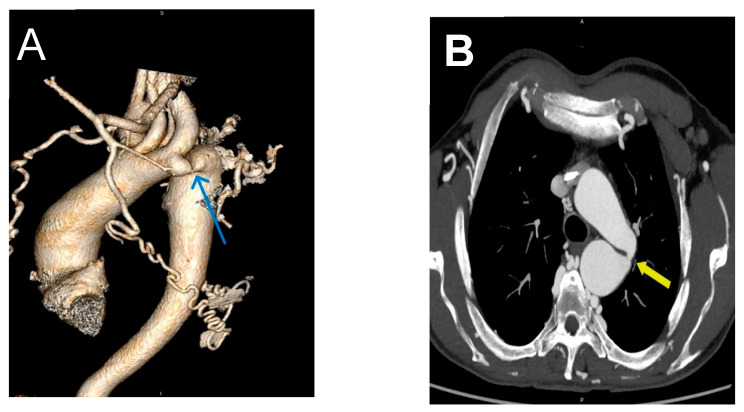
(**A**) MSCT aortography 3D reconstruction (blue arrow) and (**B**) transverse section of MSCT aortography with coarctation and lumen narrowing up to 3 mm (yellow arrow).

The left ventricle was concentrically hypertrophic and globally hypocontractile, with paradoxical movements of the septum. This results in reduced total systolic function (EF approximately 45%). Paradoxical septal motion was observed and is interpreted as a consequence of altered ventricular mechanics secondary to pressure overload, caused by aortic stenosis, and left ventricular hypertrophy. On computerized aortography, narrowing of the aorta to approximately 0.6 cm was observed after the separation of the left subclavian artery, which reduces the lumen to 3 mm (Figure 2A,B).

Several surgical approaches have been described in the literature for the treatment of adult patients with acquired heart disease and periductal coarctation of the aorta. The following staged procedures are indicated: medial sternotomy and left thoracotomy, catheter intervention of coarctation combined with sternotomy, and one-stage simultaneous correction of lesions through medial sternotomy [7].

An extra-anatomic ascending-to-descending aortic bypass has been described in earlier reports; however, the technique was further developed and popularized through larger clinical experiences, particularly by the Mayo Clinic group [8,9,10]. In 2001, Connolly et al. [11] published a series of 18 patients treated with this technique for indications of concurrent cardiac procedure or complex coarctation/recoarctation. They reported no complication such as a surgical mortality, paraplegia, or graft-related complications, along with significant improvements in systolic blood pressure after surgery [11]. Izhar et al. described the posterior pericardial approach through median sternotomy, which has become widely adopted [9].

The one-stage approach via medial sternotomy offers several distinct advantages. This technique avoids the complications of anatomical repair with recoarctation (5–30% risk) [8] and interventional repair with a 14–18% risk of acute complications, including aortic rupture/dissection [9,10]. Moreover, it also avoids or minimizes the risk of paraplegia and the need for an additional surgical sternotomy or thoracotomy with a staged procedure. Patients who have significant atherosclerotic disease of the descending thoracic aorta should not be treated with this procedure. Although the choice of the appropriate surgical approach for these patients should be individualized, a single-stage ascending–descending aortic bypass with simultaneous valve reconstruction or replacement is a safe alternative for the treatment of this set of lesions.

### 2.1. Preoperative Evaluation

Thoracic echocardiographic examination of the aorta with pressure gradient measurement across the coarctation provides a complete and reliable assessment of the anatomy and severity of the lesion. Computed tomography (CT) angiogram or magnetic resonance angiography (MRA) are non-invasive procedures for defining the anatomy and severity of coarctation and represent the gold standard in surgical planning. A gradient of 30 mmHg or greater is considered significant and an indication for correction. Surgery is also indicated for patients who present with symptoms of worsening fatigue, dyspnea on exertion, and severe hypertension requiring increased doses of anti-hypertensive medications. Additional preoperative evaluation should include a transesophageal echocardiogram to determine ventricular function and the presence of hypertrophy, as well as to examine the aortic valve and its function. Up to 40% of patients with coarctation will have a bicuspid aortic valve [4]. Cardiac catheterization with coronary angiogram should be performed in patients at risk of coronary artery disease. A complete evaluation of the thoracic aorta is critical to identify the presence of significant calcifying atherosclerotic disease in the ascending and descending thoracic aorta, which may preclude this approach. For this evaluation, we use computed tomography without contrast.

### 2.2. Operational Steps

We used intraoperative transesophageal echocardiography (TEE) to evaluate valvular pathology and confirm the absence of atheroma of the ascending and descending aorta. In addition, the TEE probe was left in place in the transgastric position to help protect the esophagus during posterior pericardial dissection and aortic mobilization. After a standard median sternotomy, the ascending aorta is fully mobilized to the aortic arch. Aortic canulation was placed high in aortic arch, under innominate artery. The common femoral artery was also cannulated to ensure adequate perfusion pressure for extracorporeal circulation (ECC). The superior and inferior vena cavae are cannulated separately to ensure adequate venous blood flow during positioning of the heart for distal graft anastomosis construction. The left ventricular vent was placed through the superior right pulmonary vein. Cardiopulmonary bypass is administered at a rate of 2.2–2.4 L/min/m^2^, with systemic cooling maintained at 32 °C. Both antegrade and retrograde cardioplegia were used for optimal myocardial protection due to ventricular hypertrophy. Local ice hypothermia was applied to the heart surface to protect the myocardium.

**Figure 3:**
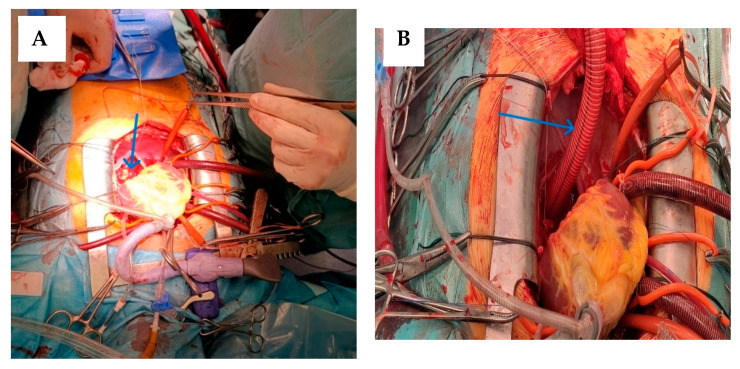
(**A**) View of the lifted heart, with the opening of the posterior pericardium (blue arrow). (**B**) Construction of anastomosis (blue arrow).

After cardioplegic arrest, the apex of the heart was then lifted from the pericardial floor to expose the posterior pericardium (Figure 3A). The descending thoracic aorta and esophagus are palpated. The pericardium was opened longitudinally, at the level of the oblique sinus, and extends from the diaphragmatic recess of the pericardium to the left inferior pulmonary vein. This was the approach to the descending thoracic aorta for the creation of distal anastomosis. Care is taken to avoid injury to the right esophagus (by palpating the TEE probe) and to the exposed sufficient length of the aorta to allow comfortable placement of a Satinsky clamp or other partial vascular occlusion.

A coil gauge is then used to estimate the diameter of the synthetic graft to be used, based on the size of the descending aorta. Typically, a 20 mm to 22 mm vascular graft is sufficient for most adults. We used a 20 mm Dacron graft. The required length of the coil was estimated using an umbilical tape, guiding the tape anterior to the inferior vena cava through the oblique sinus and around the right atrium to the right lateral ascending aorta. A coil length of 26 to 30 cm is usually sufficient in most adults. The graft was slightly angled upward to allow direction to the anterior surface of the inferior vena cava. A partial occlusion clamp is then placed on the anterior wall of the exposed descending thoracic aorta. A longitudinal aortotomy was made with Potts scissors, and an end-to-side anastomosis was constructed using 4-0 polypropylene sutures. This was the most technically difficult part of the operation, and careful and precise suture placement is essential for optimal hemostasis (Figure 3B). After the graft has been placed anterior to the inferior vena cava as described before, the clamp is placed on the mid-portion of the graft and the partial occlusion clamp is removed. Adequate hemostasis is ensured at the anastomosis, and the heart was lowered into a normal position.

**Figure 4:**
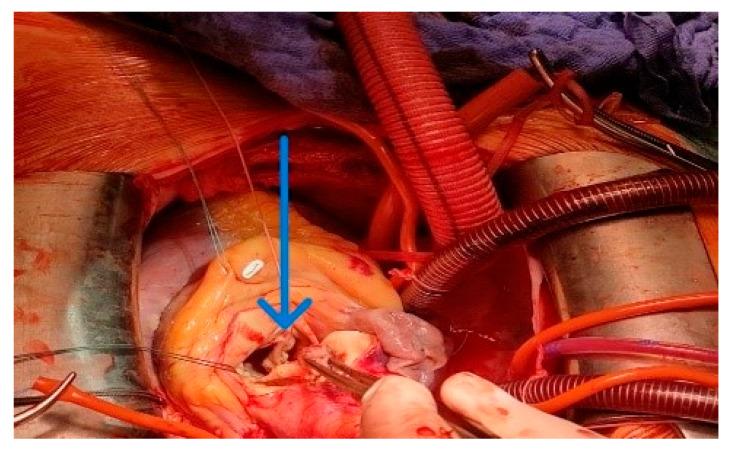
The graft is passed anterior to the inferior vena cava, the ascending aorta was opened with a transverse incision. The inspection confirmed bicuspid aortic valve type 1 (blue arrow), extraction and decalcification of the native valve was performed. Placed individual U-shaped 2-0 ethibond on the aortic annulus and the aortic valve is replaced with a mechanical prosthesis.

Then was simultaneous replaced the aortic valve with St. Jude Medical (SJM) Regent mechanical prosthesis N023 (Figure 4).

**Figure 5:**
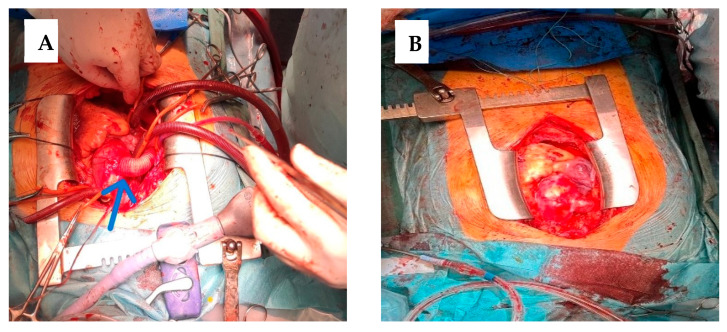
(**A**) Proximal graft (dacron graft No. 20 mm-blue arrow) anastomosis performed with Asc. Aorta. (**B**) Hemostasis after separation from ECC.

Next was the performed proximal anastomosis of the graft and ascending aorta. A longitudinal aortotomy was made at this point on the right lateral wall of the ascending aorta (identified while the aorta is filled with antegrade cardioplegia). The graft is beveled and adjusted to the appropriate length and orientation to ensure smooth flow around the right edge of the heart, taking care not to compress the right atrium with the graft. The anastomosis was formed with 4-0 polypropylene suture (Figure 5A), and the aortic root is vented through the anastomosis before removal of the aortic cross-clamp. The final graft configuration and hemostasis were illustrated in Figure 5A,B. After separation from cardiopulmonary bypass, transesophageal echocardiography was performed to confirm adequate antegrade flow through the graft and to confirm the absence of intracardiac air.

## 3. Discussion

The concomitant presence of aortic coarctation and aortic valve stenosis in adult patients represents a rare but complex surgical challenge, requiring a tailored approach to address both lesions effectively while minimizing operative risk. The strategy of performing an extra-anatomic ascending-to-descending aortic bypass combined with aortic valve replacement in a single operative session offers an elegant and physiologically sound solution for this subset of patients. This approach provides simultaneous relief of left ventricular outflow obstruction and systemic hypertension caused by the coarctation. while avoiding the technical and hemodynamic limitations of conventional anatomical repair in a high-risk setting. Izhar et al. in their study described 17 patients undergoing ascending-to-descending aortic bypass via a posterior pericardial approach, with follow-up extending up to 12 years (mean 2.7 ± 3.3 years), demonstrating no early or late mortality and no graft-related complications. Similarly, Curran et al. reported long-term outcomes in 81 consecutive patients with coarctation treated with ascending–descending aortic bypass through median sternotomy between 1985 and 2012, with a minimum follow-up of 10 years. In their series, there was no perioperative mortality, and survival at 5, 10, and 20 years was 94%, 90%, and 85%, respectively, comparable to the general population. These consistent findings across studies highlight the safety, durability, and excellent long-term outcomes of this extra-anatomic approach, particularly in complex or redo cases. Moreover, the ability to combine this procedure with intracardiac interventions further supports its role as a versatile surgical strategy [8,9].

According to the 2022 ESC/EACTS Guidelines for the management of aortic disease and the 2023 ACC/AHA Guideline for Adult Congenital Heart Disease, surgical intervention is recommended for patients with significant coarctation (gradient > 20 mmHg or evidence of collateral circulation) and for those with concomitant cardiac pathology requiring open-heart surgery [12,13,14,15]. In this context, performing a single-stage repair through a median sternotomy aligns with guideline recommendations, particularly when both lesions can be corrected effectively with one cardiopulmonary bypass run.

Historically, the standard management of adult coarctation with associated valvular pathology involved a staged approach—first addressing the aortic valve lesion via median sternotomy, followed by delayed repair of the coarctation either through left thoracotomy or via an endovascular route. Although feasible, the two-stage strategy exposes patients to cumulative surgical stress, increased risk of interval hypertension, and potential for persistent afterload mismatch with left ventricular remodeling before definitive correction of the coarctation [16,17]. Moreover, the second-stage thoracotomy or stent procedure can be technically demanding, particularly in the presence of mediastinal adhesions or calcified aortic segments.

The single-stage extra-anatomic bypass from the ascending-to-descending aorta through a posterior or anterior pericardial route was introduced to overcome these limitations [18,19]. In the present case, the graft was passed anterior to the inferior vena cava through an opened pericardium, minimizing cardiac traction and avoiding injury to posterior mediastinal structures. This modification allows optimal graft positioning and facilitates concomitant aortic valve replacement under the same cardiopulmonary bypass run. By maintaining a controlled field through a median sternotomy, the surgeon can manage both lesions without the morbidity of a separate thoracotomy.

Our patient was treated with anti-hypertensive and other medications, and the postoperative period was without complication. In a hemodynamically stable state, the patient was discharged from the clinic. Some patients may experience a paradoxical hypertensive episode up to 72 h after coarctation or bypass repair, which is thought to be due to increased sensitivity of aortic and carotid baroreceptors (“early” phase, up to 24 h after surgery) and increased circulating renin and angiotensin (“late” phase 7). This should be treated aggressively with intravenous sodium nitroprusside or a beta-blocker, with early administration of intravenous enalaprilat. The ACE1 inhibitor should be continued orally after oral medication. Up to half of patients may experience this syndrome, which usually resolves within 24–48 h.

A “late”-phase hypertensive episode may also lead to mesenteric arteritis secondary to inflammation, with possible necrosis of the small intestine. This syndrome can present with a wide range of symptoms, from mild abdominal pain to severe pain with fever, ileus, and gastrointestinal bleeding. As a result, its reported incidence varies from 7 to 28% of postoperative patients [12].

**Figure 6:**
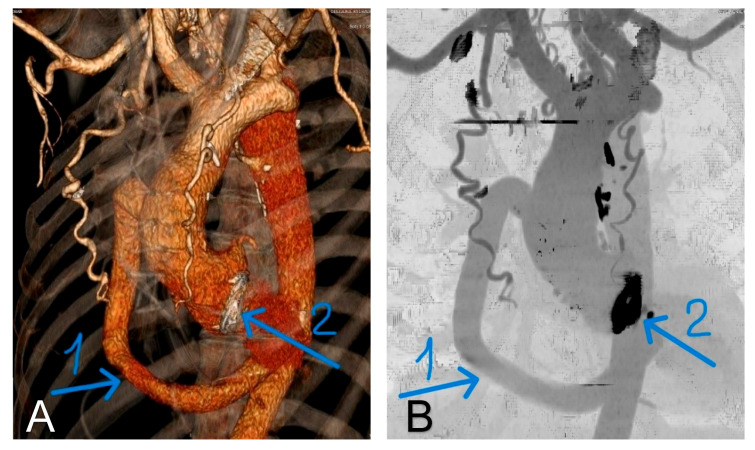
(**A**,**B**): MSCT aortography performed three months after surgery. (1) A patent extraanatomical by-pass; (2) a mechanical aortic valve prosthesis. Regression of the collateral arterial circulation between the upper and lower extremities also observed.

Follow-up with echocardiography or CT or MR aortography to re-evaluate graft status 6 months after surgery is carried out (Figure 6A,B).

Hemodynamically, the extra-anatomic bypass provides immediate pressure equalization between the ascending and descending aorta, restoring normal distal perfusion and reducing proximal hypertension [20]. Several series have demonstrated that postoperative gradients typically fall to less than 10 mmHg, with sustained long-term patency and minimal risk of graft kinking or thrombosis [21]. Furthermore, by correcting the aortic stenosis in the same session, the left ventricle is relieved of both valvular and vascular afterload, promoting early recovery of systolic function and regression of hypertrophy.

When compared with endovascular alternatives, such as covered stent implantation for coarctation followed by surgical aortic valve replacement, the single-stage surgical approach offers certain advantages. Current ESC/EACTS and ACC/AHA guidelines emphasize individualized decision-making and acknowledge that surgical repair remains preferable in cases with complex anatomy, arch involvement, or the need for concomitant cardiac procedures [13,14,22]. Endovascular treatment may be limited by anatomical constraints—particularly in patients with extensive post-stenotic dilatation, severe calcification, or arch involvement—and carries a recognized risk of aortic wall injury and late aneurysm formation [23]. Hybrid strategies, while appealing in selected patients, still necessitate two separate interventions and expose patients to the risk of contrast nephropathy, radiation, and prosthesis mismatch between procedures. In contrast, the extra-anatomic bypass achieves a definitive anatomical and functional correction under direct vision, without reliance on endovascular devices or the need for future reintervention.

Long-term outcomes of the ascending-to-descending extra-anatomic bypass have been favorable, with reported 10-year graft patency rates exceeding 90%, low incidence of reintervention, and significant improvement in systemic hypertension [24,25]. Importantly, this technique avoids the potential spinal cord ischemia associated with extensive descending aortic cross-clamping, as the distal anastomosis can be performed under partial bypass with preserved collateral flow. In our case, postoperative recovery was uneventful, with the normalization of upper–lower extremity pressure gradient and regression of left ventricular hypertrophy, consistent with previously published data.

Nevertheless, some limitations of the approach should be acknowledged. The extra-anatomic graft does not reconstruct the native isthmus and may alter flow dynamics in the arch if significant arch hypoplasia is present. Moreover, the long-term durability of the prosthetic conduit depends on patient-specific factors such as hypertension control and connective tissue quality. Regular imaging surveillance is therefore mandatory to detect potential graft dilatation or anastomotic pseudoaneurysm formation [26].

## 4. Conclusions

Extra-anatomic ascending-to-descending aortic bypass has been confirmed as a safe and effective technique in adults with coarctation over the past 5 years. This procedure often allows a single operative approach simultaneous with valve replacement or repair.

Long-term complications, such as pseudoaneurysm or degenerative graft loss, require lifelong radiological monitoring. Combined cases with multiple cardiac procedures are an increasingly common indication for this approach, with favorable outcomes.

## Data Availability

The data presented in this study are available within the article.
